# Comprehensive analysis of genomic mutation signature and tumor mutation burden for prognosis of intrahepatic cholangiocarcinoma

**DOI:** 10.1186/s12885-021-07788-7

**Published:** 2021-02-03

**Authors:** Rui Zhang, Qi Li, Jialu Fu, Zhechuan Jin, Jingbo Su, Jian Zhang, Chen Chen, Zhimin Geng, Dong Zhang

**Affiliations:** Department of Hepatobiliary Surgery, The First Affiliated Hospital of Xi’an Jiaotong University, Xi’an, 710061 China

**Keywords:** Genomic mutation signature, Tumor mutation burden, Intrahepatic cholangiocarcinoma, Prognostic biomarker, Nomogram

## Abstract

**Background:**

Intrahepatic cholangiocarcinoma (iCCA) is a highly lethal malignancy of the biliary tract. Analysis of somatic mutational profiling can reveal new prognostic markers and actionable treatment targets. In this study, we explored the utility of genomic mutation signature and tumor mutation burden (TMB) in predicting prognosis in iCCA patients.

**Methods:**

Whole-exome sequencing and corresponding clinical data were collected from the ICGC portal and cBioPortal database to detect the prognostic mutated genes and determine TMB values. To identify the hub prognostic mutant signature, we used Cox regression and Lasso feature selection. Mutation-related signature (MRS) was constructed using multivariate Cox regression. The predictive performances of MRS and TMB were assessed using Kaplan–Meier (KM) analysis and receiver operating characteristic (ROC). We performed a functional enrichment pathway analysis using gene set enrichment analysis (GSEA) for mutated genes. Based on the MRS, TMB, and the TNM stage, a nomogram was constructed to visualize prognosis in iCCA patients.

**Results:**

The mutation landscape illustrated distributions of mutation frequencies and types in iCCA, and generated a list of most frequently mutated genes (such as *Tp53, KRAS, ARID1A,* and *IDH1*). Thirty-two mutated genes associated with overall survival (OS) were identified in iCCA patients. We obtained a six-gene signature using the Lasso and Cox method. AUCs for the MRS in the prediction of 1-, 3-, and 5-year OS were 0.759, 0.732, and 0.728, respectively. Kaplan–Meier analysis showed a significant difference in prognosis for patients with iCCA having a high and low MRS score (*P* < 0.001). GSEA was used to show that several signaling pathways, including MAPK, PI3K-AKT, and proteoglycan, were involved in cancer. Conversely, survival analysis indicated that TMB was significantly associated with prognosis. GSEA indicated that samples with high MRS or TMB also showed an upregulated expression of pathways involved in tumor signaling and the immune response. Finally, the predictive nomogram (that included MRS, TMB, and the TNM stage) demonstrated satisfactory performance in predicting survival in patients with iCCA.

**Conclusions:**

Mutation-related signature and TMB were associated with prognosis in patients with iCCA. Our study provides a valuable prognostic predictor for determining outcomes in patients with iCCA.

**Supplementary Information:**

The online version contains supplementary material available at 10.1186/s12885-021-07788-7.

## Background

Cholangiocarcinoma (CCA) is a highly lethal and aggressive malignancy originating from the biliary epithelium. Based on the anatomical site of origin, CCA can be classified into three subtypes including perihilar (pCCA), distal (dCCA), and intrahepatic (iCCA) [1]. iCCA accounts for 10–20% of primary liver cancers and approximately 20% of biliary-tract cancers [2]. Postoperative 5-year overall survival (OS) in iCCA patients is poor (30–40%) [3, 4]. In the last decade, iCCA has shown an increasing incidence rate and mortality worldwide, which contrasts with the decreasing trends shown by pCCA and dCCA [5]. Surgical resection is currently the mainstay of curative-intent treatment for patients in the early stage; however, the vast majority of patients miss the opportunity for radical surgery [6, 7]. Nevertheless, even after resection, iCCA patients show a high incidence of recurrence [8–10]. These findings highlight the importance of discovering novel prognostic biomarkers and constructing predictive models for patients with iCCA. Such approaches can be used to make treatment determinations and improve patient prognosis.

Cancer is often accompanied by an accumulation of various genetic mutations. Accumulated somatic mutations contribute to tumorigenesis and progression of malignancy. Genetic mutations are consistently present and critical factors that determine gene function and biological behavior in malignant tumors [11, 12]. Certain genetic mutations may be used as prognostic indicators to predict patient survival and response to adjunctive therapy [13–16]. Several frequently occurring genetic alterations, including *TP53, KRAS, ARID1A, IDH1/2, BAP1,* and *PBRM1*, have been identified in iCCA [17, 18]. However, the prognostic implications of these somatic alterations in iCCA are poorly understood. Improving our understanding of these genetic mutations is vital for selecting prognostic genetic biomarkers, identifying high-risk CCA patients harboring pertinent genetic mutations, and tailoring treatment strategies in clinical practice.

Tumor mutation burden (TMB) is defined as the number of somatic (such as missense, deletion, or insertion) mutations per megabase of genome examined [19]. Recent studies have indicated that TMB may be used as a biomarker to predict patient response to immune checkpoint inhibitor (ICI) therapy [20, 21]. Furthermore, numerous studies have shown that TMB can be used to predict the effectiveness of immunotherapy against various cancers such as non-small cell lung carcinoma (NSCLC) [22, 23], melanoma [24], esophagogastric [25], and colorectal [26]. Although the utility of TMB in predicting the effectiveness of ICIs has been established, few studies have investigated the prognostic potential of TMB in predicting the survival of patients with iCCA.

Whole exome sequencing (WES) is regarded as the gold standard for assessing TMB values. In recent years, bioinformatic WES resources have become available from public databases, such as the International Cancer Genome Consortium (ICGC, https://dcc.icgc.org/) and cBioPortal (https://www.cbioportal.org/), enabling large-scale genomic integration and comprehensive bioinformatics analysis of various cancers. These public databases can be used to determine factors that influence anti-cancer immunotherapy.

In this study, we used WES data from ICGC and cBioPortal database to investigate the mutational landscape of iCCA; explore the potential impact of mutation-related signature on patient survival; and establish a reliable nomogram model based on mutant gene signature, TMB, and other clinical characteristics to predict the OS of patients with iCCA. The findings obtained in our present study, as well as our nomogram model, can be used to explore new potentially prognostic biomarkers and provide therapeutic targets for the treatment of patients with iCCA.

## Methods

### Collection of mutation data

**Fig. 1:**
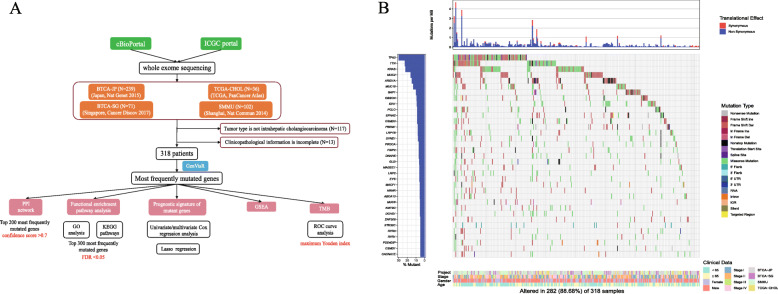
Mutational landscape of iCCA. **a** Schematic view of patient cohorts and experimental pipeline used to analyze study data; **b** Top 35 frequently mutated genes are shown in the waterfall plot

Data on somatic mutations and the corresponding clinicopathological characteristics of iCCA patients were acquired from cBioPortal (http://www.cbioportal.org) and the ICGC portal (http://dcc.icgc.org/releases/current/Projects). We selected the WES dataset for iCCA patients only. The repositories used were BTCA-JP (Japan, Nat Genet 2015) [27], BTCA-SG (Singapore, Cancer Discov 2017) [2], TCGA-CHOL (TCGA, PanCancer Atlas) [12] and Intrahepatic Cholangiocarcinoma (Shanghai, Nat Commun 2014) [28]. Only patients from these datasets with complete clinicopathological information were included. Clinical characteristics included age, gender, TNM stage, survival status, and survival time. Perl scripts were then used to extract the somatic mutation data on iCCA. The “GenVisR” and “karyoploteR” functions in the R software package were used to visualize the genetic landscape. Comparison of these mutations with those listed in the Cancer Hotspot Mutation database (https://www.cancerhotspots.org/#/home) provided information on whether these mutations are predicted to be putative driver mutations. We also screened the top mutations by comparing them with the data available in OnkoKB (https://www.oncokb.org/) to determine whether any of these mutations are known to be clinically associated. We then used the “corrplot” package to explore correlations between mutations that co-occurred or were exclusive of each other. In order to show differences in mutation across different ethnic groups, we also used the “yyplot,” “ggplot2,” and “ggord” package to perform the principal component analysis (PCA). Finally, a carton workflow was plotted to describe our data/pipeline (Fig. 1a).

### Construction of protein-protein interaction (PPI) network

We selected the top 200 most frequently mutated genes to investigate the possible interactive relationships between these genes. These genes were inputted into the STRING database (https://stringdb.org/) to generate the PPI network; a confidence score > 0.7 was designated as the cut-off criterion. We also modified the PPI network using Cytoscape. Then, mutated gene nodes with an edge of > 5 were extracted as the most important targets.

### Functional enrichment pathway analysis

The top 300 most frequently mutated genes were selected to perform an enrichment pathway analysis. The “org. Hs.eg.db,” “ggplot2,” “clusterProfiler,” and “enrichplot” packages were utilized for Gene Ontology (GO) analysis and analysis of the Kyoto Gene and Genome Encyclopedia (KEGG) pathways; FDR < 0.05 was considered to be statistically significant.

### Hub prognostic mutant genes and construction of the prognostic model

Next, we used the univariate Cox regression analysis to screen the hub mutated genes for use in iCCA prognosis. We performed a dimensionality reduction analysis of survival-associated mutant genes using the least absolute shrinkage and selection operator (Lasso) regression, and “survival” and “glmnet” packages in R. Lasso sub-selects prognostic mutant genes by imposing a penalty proportional to the contraction of the regression coefficient. We then performed a multivariate Cox regression analysis to establish the mutation related signature (MRS), which was calculated using the following formula:

$$ \mathrm{MRS}={\sum}_{i=1}^n\left({\upbeta}_i\ast {\mathrm{Mut}}_i\right), $$

where β_i_ is the coefficient and Mut_i_ represents the mutation status of genes (if the status is Mutation, Mut_i_ = 1; if the statue is Wild, Mut_i_ = 0). Subsequently, the 318 iCCA patients were classified into low and high groups according to the median MRS. OS was estimated to compare survival between the two groups, with *P* value < 0.05 indicating a significant difference. Receiver operating characteristic (ROC) curves were generated to evaluate the performance of MRS in predicting the 1-, 3-, and 5-year survival. Forest plots were also used to show the hazard ratio (HR) of selected prognostic mutated genes using the “survminer” package.

### TMB values of patients with iCCA: estimation and prognostic analysis

TMB was defined as total number of mutations per coding area. All of the non-synonymous variants in the coding region were counted, and silent mutations were not considered. Genomic mutations for the 318 iCCA patients were specifically extracted. Because 38 Mb is routinely used as total exon length in humans, we calculated TMB as total mutation frequency divided by 38 [29]. The TMB of each patient was calculated using this method, and corresponding survival data were merged. Then, we divided the patients into high- and low-TMB groups according to the cut-off TMB values, which were determined using the maximum Youden index of the ROC curve.

### Gene set enrichment analysis (GSEA)

To examine the activity of potentially involved biologic pathways in the high- and low-group of patients based on MRS and TMB, we performed GSEA (MSigDB; version 7.1) using the “KEGG,” “GO,” and “immunologic signatures” gene sets from the Molecular Signature Database. The mutation gene list for the assessment of MRS and TMB status was used as input phenotype data. The analysis was performed 1000 times for gene-set permutations, and pathways with *P* < 0.05 were considered statistically significant.

### Statistical analysis

All statistical analyses were conducted using SPSS 24.0, R software (version 4.0.2), and GraphPad Prism 8.0. Student’s t-test was used to compare continuous variables, and the χ^2^ test or Fisher’s exact test was used to compare categorical data. The effects of AJCC TMN stage, MRS, and TMB on survival were assessed using the log-rank test and Kaplan–Meier method. Multivariable Cox regression analysis was used to determine independent risk factors. Fisher’s exact test was used to analyze the correlations between gene mutations that co-occurred or were exclusive of each other. A nomogram model was then constructed, and predictive performance of the nomogram was estimated using C-index and calibration plot.

## Results

### Landscape of genetic mutation profiles in iCCA

The demographic and clinicopathological characteristics of 318 patients with iCCA, examined using WES, are listed in Table 1. Our cohort included 192 men and 126 women. Median age at the time of diagnosis was 62 years (range, 26–89 years). Our results revealed that 15 genes (*TP53*, *TTN*, *KRAS*, *MUC2*, *ARID1A*, *MUC16*, *BAP1*, *OBSCN*, *CSMD3*, *EPHA2*, *IDH1*, *PCLO*, *LRP1B*, *PBRM1*, and *SYNE1*) were mutated in more than 20 samples. The genetic mutation frequency is shown in the Supplementary Table 1. We visualized the landscape of mutation profiles using the “GenVisR” package, which shows only the top 35 most frequently mutated genes across the 318 samples (Fig. 1b). Moreover, we mapped the mutated genes whose mutation frequency was more than 5 on the chromosomes; in this map, the color red indicates the high-frequency mutation sites (Supplementary Fig. 1A). In addition, we provided a lollipop plot for the top five mutated genes (*TP53, KRAS, MUC2, ARID1A,* and *MUC16*) as Supplementary Fig. 1B. We also explored the correlation between mutations that co-occurred or were exclusive of each other (Supplementary Fig. 2A). Our results indicate that *IDH1* was recurrent with BAP1/PBRM1/ARID1A/PIK3CA, but exclusive of *KRAS/TP53/MUC2*. We also conducted a PCA to examine our cohort of iCCA patients with respect to their different ethnic backgrounds. Our results indicate no difference in mutational pattern between different ethnic populations (Supplementary Fig. 2B). We compared the mutations occurring in our patient cohort with those listed in the Cancer Hotspot Mutation database, and found that 8 (*TP53, KRAS, ARID1A, BAP1, IDH1, PBRM1, PIK3CA,* and *KMT2D*) of the top 35 mutations were predicted to be putative driver mutations. We also screened the top mutations to find actionable genes on the OnkoKB website. Our findings indicate that three genetic mutations (*IDH1, KRAS,* and *PIK3CA*) were potentially viable molecular alterations.

**Table 1:** Clinical characteristics of patients from ICGC and cBioPortal database

Variables	All patients (***n*** = 318)	
	Number (n)	Percent (%)
Age, years		
Median	62	
Range	26–89	
< 65	178	56.0
≥ 65	140	44.0
Gender		
Female	126	39.6
Male	192	60.4
TNM Stage		
Stage I	80	25.2
Stage II	94	29.6
Stage III	37	11.6
Stage IV	107	33.6
Project		
TCGA-CHOL	32	10.0
BTCA-JP	136	42.8
BTCA-SG	48	15.1
SMMU	102	32.1
TMB, mut/Mb		
Median	1.25	
Range	0.03–54.74	
< 10	306	96.2
≥ 10	12	3.8

### PPI network of mutant genes

**Fig. 2:**
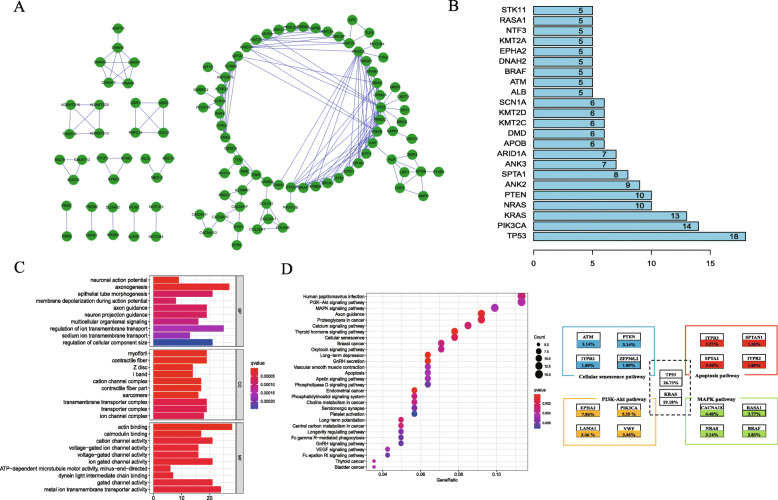
Protein to protein interaction (PPI) network and functional enrichment pathway of mutated genes. **a** PPI network of mutated genes; **b** nodes with an edge of > 20 were considered hub genes; **c** Gene Ontology analysis. Top 30 enriched terms in GO. Cellular component (CC); biological process (BP); molecular function (MF). **d** KEGG analysis of mutated genes. The 30 significantly enriched KEGG pathways. Circle size indicates gene numbers, and color represents adj. *P*-value. Frequencies of major genetic mutations in the enriched pathways are shown

The top 200 most frequently mutated genes were selected to construct a protein-protein interaction network (PPI). We performed a PPI network-based analysis using STRING database to determine whether the mutated genes functionally interacted with each other and were involved in tumorigenesis. The networks were also visualized using Cytoscape, which contained 108 nodes and 182 interacting pairs (Fig. 2a). The top hub genes with the highest clustering included *TP53, PIK3CA, KRAS, NRAS, PTEN, ANK2, SPTA1, ANK3,* and *ARID1A* (Fig. 2b).

### Functional pathway analysis of hub mutant signature

We used the R software package to perform a functional pathway analysis of the top 300 mutant genes. Figure 2c shows the top 30 enriched GO terms associated with regulation of signaling in multicellular organisms, ion transmembrane transport, and transmembrane transporter complex and ion-gated channel activity. In addition, KEGG pathway analysis demonstrated enrichment of mutant genes in several signaling pathways involved in malignancy, such as the PI3K-AKT, MAPK, proteoglycan, and calcium-signaling pathways. We also expanded the pathway to include major genes from the enriched pathways (PI3K-AKT, MAPK, Cellular senescence and apoptosis pathway) (Fig. 2d).

### Prognostic signature of mutant genes

**Fig. 3:**
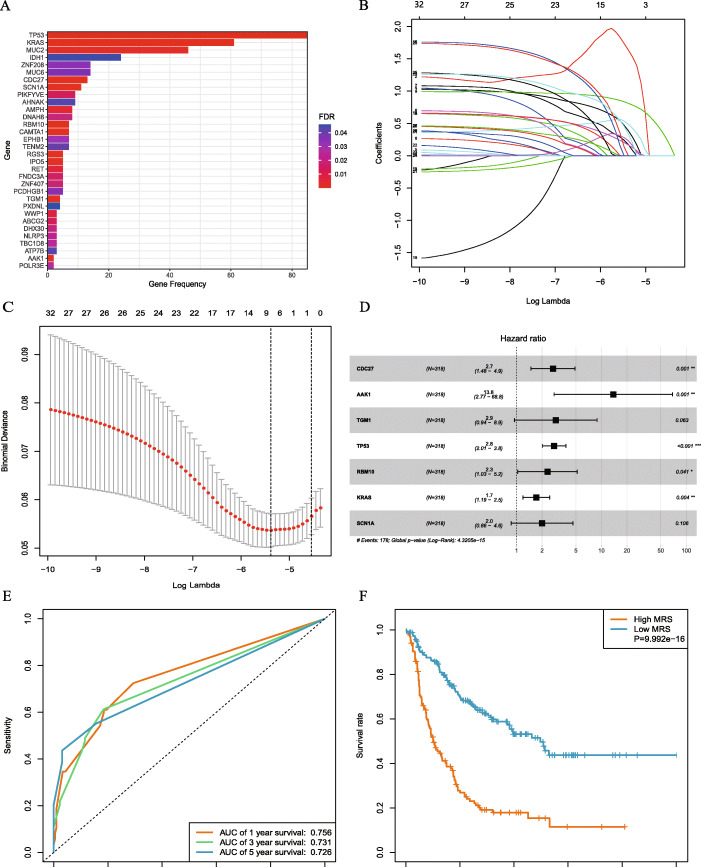
Lasso regression was used to screen the MRS for the predictive model; **a** a bar plot shows 32 mutant genes significantly associated with OS; **b** Lasso coefficient profiles of mutated genes in our iCCA cohort. **c** A coefficient profile plot was generated to find the optimal parameter (lambda). **d** Hazard ratios (HR) for the selected prognostic mutated genes were generated using multivariate Cox analysis and are shown in Forest plots; **e** KM plotter shows differences between the high- and low-MRS groups, indicating that high MRS was associated with poor survival outcomes. **f** Time-dependent ROC curve analysis shows that the AUCs for MRS were 0.759, 0.732, and 0.728 for 1, 3, and 5-year OS, respectively; this analysis demonstrates the satisfactory predictive accuracy of the MRS model

To explore the prognostic roles of gene mutations in iCCA, we used univariate Cox regression to analyze patient survival. Patients were categorized into wild-type and mutant-type according to their gene-mutation status. Thirty-two mutated genes, significantly associated with OS, were obtained, and the Kaplan-Meier analysis was used to assess their prognostic value (Fig. 3a, Supplementary Fig. 3 and 4). Furthermore, we obtained a gene signature for 12 prognostic mutated genes using the Lasso Cox method (Fig. 3b and c). We further utilized multivariate Cox regression analysis to establish a model that included six mutated genes to predict the survival of patients with iCCA (Fig. 3d). Using multivariate Cox regression analysis, regression coefficients were weighted for the six mutant genes to establish a risk-prediction model. MRS was calculated as follows: MRS = (0.9772 × CDC27) + (3.3262 × AAK1) + (1.0356 × TP53) + (0.8040 × RBM10) + (0.5645 × KRAS) + (1.4581 × IPO5) (Table 2). Based on the MRS value, patients were divided into the high- and low-risk groups. Patients in the low-risk group showed improved survival compared with that of patients in the high-risk group (*P* < 0.001; Fig. 3e). Our results indicate that using MRS to predict 1-, 3-, and 5-year survival yielded an AUC value of 0.759, 0.732, and 0.728, respectively (Fig. 3f), indicating high prediction efficiency.

**Table 2:** 6- mutation gene risk signature from multivariable Cox regression analysis

Gene	Coefficient	HR	95% CI	P-value
CDC27	0.9772	2.657	1.463–4.826	0.001333
AAK1	3.3263	27.835	6.606–117.292	5.83e-06
TP53	1.0356	2.817	2.050–3.871	1.70e-10
RBM10	0.8040	2.235	1.001–4.987	0.049659
KRAS	0.5645	1.759	1.227–2.520	0.002095
IPO5	1.4581	4.298	1.743–10.600	0.001547

Abbreviations: *CI* confidence interval; *HR* hazard ratio

### Prognostic impact of TMB and TNM stage in patients with iCCA

**Fig. 4:**
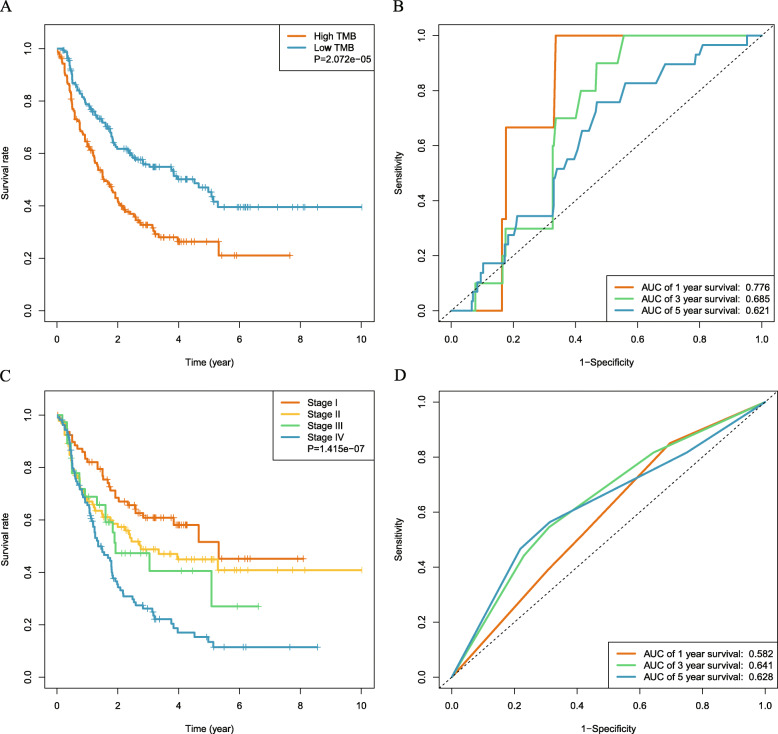
Prognostic ability of TMB in predicting the OS of iCCA patients. **a** Kaplan–Meier plot, used to analyze the difference in survival of the high- and low-TMB group, shows that the high-TMB group had poor survival outcomes. **b** Time-dependent ROC curve analysis shows that the AUCs for TMB were 0.776, 0.685, and 0.621 for 1, 3, and 5-year OS, respectively; this analysis demonstrates the satisfactory predictive accuracy of the TMB model. **c** Kaplan–Meier plot shows significant differences between patients grouped with respect to their TNM stage. **d** Time-dependent ROC curve analysis of the TNM stage

Next, we analyzed the utility of TMB in prognosis. Median TMB was 1.29 mutations/Mb (range, 0.03–54.74 mutations/Mb). We then analyzed the predictive performance of TMB with respect to OS. AUC of the ROC curve for TMB with respect to 1-, 3-, and 5-year survival was 0.776, 0.685, and 0.621, respectively (Fig. 4b). TMB threshold values were calculated using the 3-year ROC curve analysis with maximum Youden index. We found that maximal AUC value was achieved when the cut-off value of TMB was 1.29. Therefore, we defined 1.29 mutations/Mb as the cut-off value, classifying TMB > 1.29 mutations/Mb as the high group (*N* = 157) and TMB ≤ 1.29 mutations/Mb as the low group (*N* = 161). KM plotter of survival analysis showed that OS was significantly decreased in patients with high TMB compared with those having low TMB (*P* < 0.001; Fig. 4a). We also explored the relationship between TMB and prognostic mutant genes. Our results indicate that TMB was moderately correlated with *PIKFYVE* (*r* = 0.31) and *RGS3* (*r* = 0.34) (Supplementary Fig. 5). In addition, we explored the prognostic role of TNM stage in iCCA. Our results demonstrate that TNM stage was significantly correlated with OS; however, AUC of the TNM stage in predicting 1-, 3-, and 5-year survival was 0.582, 0.641, and 0.628, respectively, indicating a poor prognostic performance compared with those of MRS and TMB (Fig. 4c and d).

### Gene set enrichment analysis

**Fig. 5:**
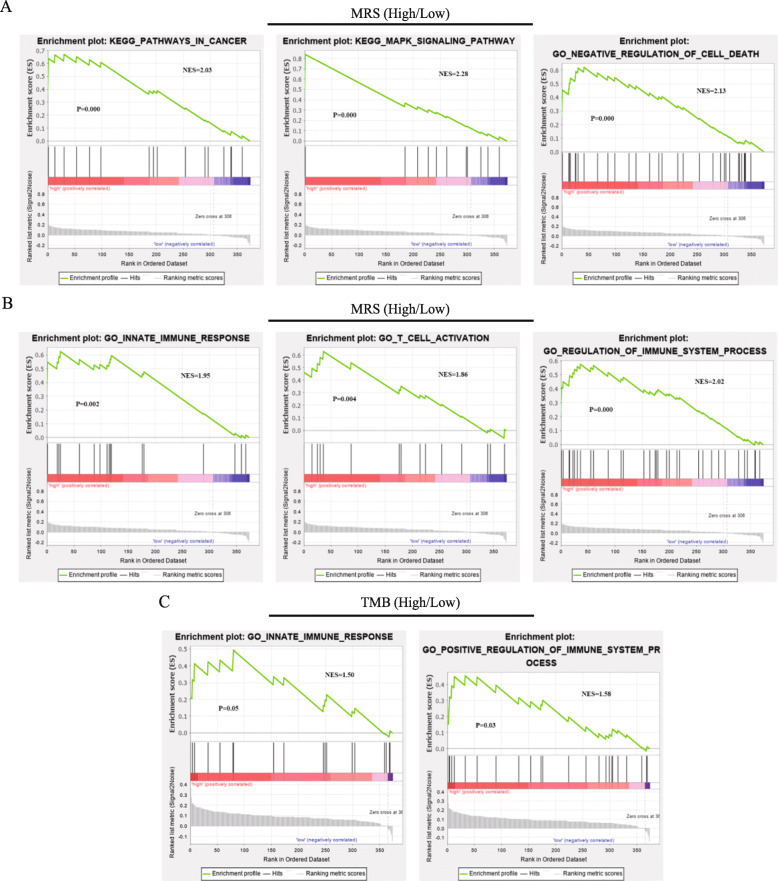
Functional analysis of different MRS and TMB groups conducted using gene set enrichment analysis (GSEA). **a** and **b** Representative KEGG pathways and GO pathways analyzed in the low-MRS versus high-MRS group. **c** Representative KEGG pathways and GO pathways analyzed in the low-TMB versus high-TMB group

We used GSEA to visualize the enriched biological processes in the different MRS and TMB groups. Our results indicate that patients in the high MRS group were prone to show associations with the innate immune response, negative regulation of cell death, positive regulation of immune system processes, T-cell activation, MAPK signaling pathway, and cancer pathways (Fig. 5a and b). The high TMB group was enriched in positive regulation of immune-system processes and cancer pathways. These results demonstrate that crosstalk involved in tumor-signaling pathways and immune-system processes was upregulated in patients with high MRS or TMB (Fig. 5c).

### Construction and evaluation of the nomogram

**Fig. 6:**
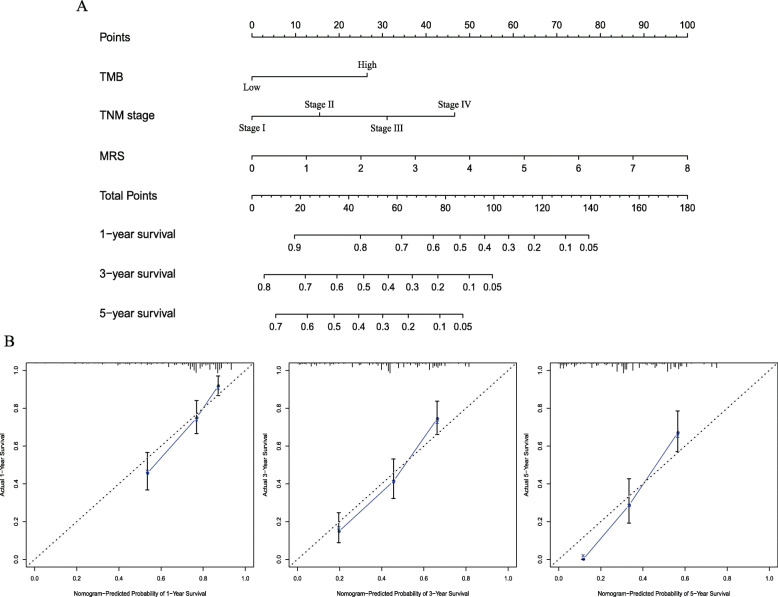
Construction of prognostic nomogram for patients with iCCA. **a** The predicted 1-, 3-, and 5-year survival rates in iCCA patients based on our nomogram that included MRS, TMB, and TNM stage. **b** Calibration plots show the concordance between predicted and actual observation and prediction in 1, 3, and 5-year OS

To find the independent prognostic biomarkers, we used univariate Cox regression analysis to analyze the associations between OS and factors such as MRS, age, sex, TNM stage, and TMB (Table 3). The results of multivariate Cox regression analysis demonstrated that TNM stage, MRS, and TMB were independent-risk predictors for iCCA (Table 3). We constructed a predictive nomogram based on these risk factors, which included MRS, TMB, and TNM stage (Fig. 6a). The C-index value for the nomogram was 0.721 (95% CI, 0.613–0.829). Calibration curve indicated that the observed and predicted values were consistent in predicting OS (Fig. 6b).

**Table 3:** Univariable and multivariable analysis of overall survival

	Univariable			Multivariable		
	HR	95% CI	P-value	HR	95% CI	***P***-value
**Age, years**						
< 65						
≥ 65	0.869	0.643–1.175	0.362			
**Gender**						
Female						
Male	0.686	0.787–1.439	0.686			
**TNM Stage**						
I			0.000			0.000
II	1.470	0.938–2.305	0.093	1.717	1.092–2.698	0.019
III	1.743	0.987–3.079	0.055	2.078	1.172–3.685	0.012
IV	2.716	1.799–4.101	0.000	2.963	1.957–4.485	0.000
**TMB, mut/Mb**						
Low						
High	1.967	1.454–2.660	0.000	1.500	1.085–2.073	0.014
**MRS**						
Low						
High	2.771	2.035–3.773	0.000	2.448	1.758–3.409	0.000

Abbreviations: *CI* confidence interval; *HR* hazard ratio

## Discussion

In this study, we explored the role of mutational signature and TMB in predicting the survival of patients with iCCA. First, whole exome sequencing (WES) data on iCCA were obtained from two public databases (ICGC and cBioportal), and frequently mutated genes were identified. Next, univariate, Lasso, and multivariate Cox regression analyses were used to screen for hub prognostic mutant signature and establish a mutation-risk model for predicting prognosis. After the prognostic role of MRS was confirmed, we used PPI, GO, KEGG, and GSEA analyses to reveal the potential cancer-related crosstalk involved. We also found that increased TMB was associated with poor prognosis. Furthermore, MRS, TMB, and TNM stages were confirmed as independent predictors for overall survival (OS) of patients with iCCA. Next, based on the risk factors affecting OS, we constructed a reliable nomogram model that demonstrated a satisfactory performance in predicting OS in patients with iCCA.

Gene mutations are ubiquitous in tumorigenesis and development of iCCA. Previous studies have reported comprehensive molecular alterations in biliary tract cancers [18, 28]. In our study, the most relevant mutation was *TP53* (26.7%), followed by *TTN* (20.7%), *KRAS* (19.1%), *MUC2* (14.5%), and *ARID1A* (12.9%), which was consistent with the findings of Cao et al. [30]. A study, based on approximately 500 patients with iCCA, used the three most recurrently mutated genes (*IDH1, KRAS* and *TP53*) to stratify patients into four subgroups, revealing distinct genomic and molecular features [31]. In our present study, we also found that several mutated genes, such as *IDH1* (7.5%), *BAP1* (9.1%), *PBRM1* (7.2%), and *EPHA2* (7.9%), were particular to iCCA. Jiao et al. performed exome sequencing on iCCA tissue samples, and found that frequently mutated genes (such as *BAP1*, *ARID1A*, and *PBRM1*) are involved in the chromatin-remodeling pathway. Our results on the mutational frequency of chromatin-remodeling family genes in iCCA are consistent with those reported in previous studies [32]. We investigated the possible link between genetic alternations and patient prognosis, and found that the outcomes of patients with certain genetic mutations were worse than those of wild-type patients; statistically, this finding showed borderline significance. Based on these prognostic factors, we developed a mutation risk score to predict survival. The MRS of our model was calculated based on 6 hub prognostic mutant genes (*CDC27, AAK1, TP53, RBM10, KRAS,* and *IPO5*). This MRS model showed high predictive accuracy for OS, was a reliable tool in predicting prognosis, and can be used in clinical practice. We next performed a functional pathway and GSEA analyses to uncover the molecular mechanisms underlying iCCA. Our functional enrichment pathway analysis indicated that the prognostic mutated genes were closely correlated with cancer-associated signaling pathways, such as cancer development and immune related pathway. GSEA also showed that the high MRS group was enriched in signaling involved in immune-related pathways.

In recent years, various types of immune checkpoint inhibitor (ICI) therapy have been developed for the treatment of patients with advanced-stage cancers. However, only a minority of patients benefit from ICI therapies. TMB, a novel predictive biomarker, can predict a clinical response to ICI and can be used to identify patients likely to benefit from these therapies [19]. Increased TMB indicates increased levels of tumor antigen, which is beneficial for activating the body’s immune response. Previous studies on TMB mostly focused on its capacity to predict the effectiveness of ICIs, showing a robust correlation between increased TMB and improved response to ICI therapy. However, few studies have explored the prognostic value of TMB in predicting the survival of patients with iCCA. Numerous studies have shown a relationship between TMB and survival in patients with cancer. Owada-Ozaki et al. found that increased TMB is correlated with decreased disease-free survival in NSCLC patients [33]. A study from China demonstrated that in HCC patients who had undergone radical resection, patients with increased TMB tend to show increased risk for recurrence; additionally, they also showed that TMB is an independent risk factor for RFS [34]. We show that the median TMB value in our iCCA patients was 1.25 (range 0.03–54.74). A large-scale examination of TMB in iCCA patients was performed by Cao et al. [30]. They used comprehensive genomic profiling to analyze the frequency and type of genetic aberrations, and did not observe genomic heterogeneity between Asian and Caucasian patients with iCCA; however, the relationship between TMB and prognosis was not evaluated in that study. Tian et al. investigated the genomic features of Chinese patients with iCCA, and explored the relationship between TMB and certain genetic changes [35]. It should be noted that the TMB of their cohort was greater than that determined in our study. This occurred because we only counted the non-synonymous variants.

In agreement with previous findings on other tumor types, our results show that increased TMB was correlated with poor patient prognosis. Therefore, we concluded that TMB had divergent prognostic impact in different patients with iCCA. In addition, our results indicate that a prognostic model incorporating TMB will likely improve prognostication and risk stratification in patients with iCCA.

In this study, we explored the prognostic role of MRS and TMB in patients with iCCA. Our findings indicate that the prognostic performance of the predictive model incorporating TMB or MRS was better than that of the TNM stage. Furthermore, results of our multivariate analysis indicate that TMB, MRS, and TNM stage were independent prognostic factors in iCCA. Despite these novel findings on the prognostic value of MRS and TMB, our study had several limitations. First, mutation data on iCCA were extracted from public databases that only included samples that had undergone WES. Targeted sequencing data were not used in our study. Additional WES data from patients with iCCA should be incorporated to reduce selective bias. Second, the mechanisms underlying the prognostic capability of MRS and TMB in iCCA should be further investigated. Additional experiments, performed both in vitro and in vivo, are required to support the results obtained in our present study. Finally, this study did not show whether specific mutations led to abnormal gene expression, and this question requires further investigation.

## Conclusion

In summary, our study demonstrates that mutational signature and TMB were associated with prognosis in patients with iCCA. We visualized the mutational landscape and summarized the most commonly mutated genes. We also developed a risk model based on the prognostic utility of mutated genes, and found that MRS and TMB, included in the model, had divergent prognostic impacts in patients with iCCA. Based on independent risk factors, such as TNM stage, MRS, and TMB, we then constructed a reliable nomogram model for predicting OS in iCCA patients. The nomogram developed in this study can be incorporated into the methodology used for prognostication in patients with iCCA.

## Supplementary Information

**Additional file 1 Figure S1** The specific locations and types of gene mutations on iCCA patients. **(a)** Map shows specific locations of mutations on chromosomes. Green color indicates low-frequency mutation sites, while red represents high frequency; **(b)** lollipop plot of top five mutated genes.

**Additional file 2 Figure S2** Correlation of gene mutations and PCA of iCCA patients based on different ethnic backgrounds. **(a)** Correlation of gene mutations that co-occurred or were exclusive of each other. (b) PCA of iCCA patients based on different ethnic backgrounds.

**Additional file 3 Figure S3** and **Figure S4** Kaplan–Meier plots of the 32 mutant genes significantly associated with OS.

**Additional file 4 Figure S5** Relationship between TMB and prognostic mutant genes.

**Additional file 5.**

## Data Availability

All WES data and clinical information used in this study were acquired from the ICGC portal (http://dcc.icgc.org/releases/current/Projects) (up to June 10, 2019) and cBioPortal (http://www.cbioportal.org) (up to June 10, 2019).

## Abbreviations

iCCA: Intrahepatic cholangiocarcinoma
TMB: Tumor mutation burden
MRS: Mutation related signature
ICI: Immune checkpoint inhibitor
WES: Whole exome sequencing
ICGC: International Cancer Genome Consortium
GO: Gene Ontology
KEGG: Kyoto Gene and Genome Encyclopedia
Lasso: Least absolute shrinkage and selection operator regression
GSEA: Gene set enrichment analysis
PPI: Protein-protein interaction
HR: Hazard ratio
BP: Biological process
MF: Molecular function
CC: Cellular component
KM: Kaplan–Meier
ROC: Receiver operating characteristic
AUC: Area under the curve
CDC27: Cell division cycle 27
AAK1: AP2 associated kinase 1
TP53: Tumor protein P53
RBM10: RNA binding motif protein 10
IPO5: Importin 5
HCC: Hepatocellular carcinoma
NSCLC: Non-small cell lung carcinoma
AJCC: American Joint Committee on Cancer
