# Supplementary figures and images for: Comprehensive analysis of genomic mutation signature and tumor mutation burden for prognosis of intrahepatic cholangiocarcinoma
Source: BMC Cancer. 2021 Feb 3;21:112. doi: 10.1186/s12885-021-07788-7 (PMC7860034; doi:10.1186/s12885-021-07788-7)

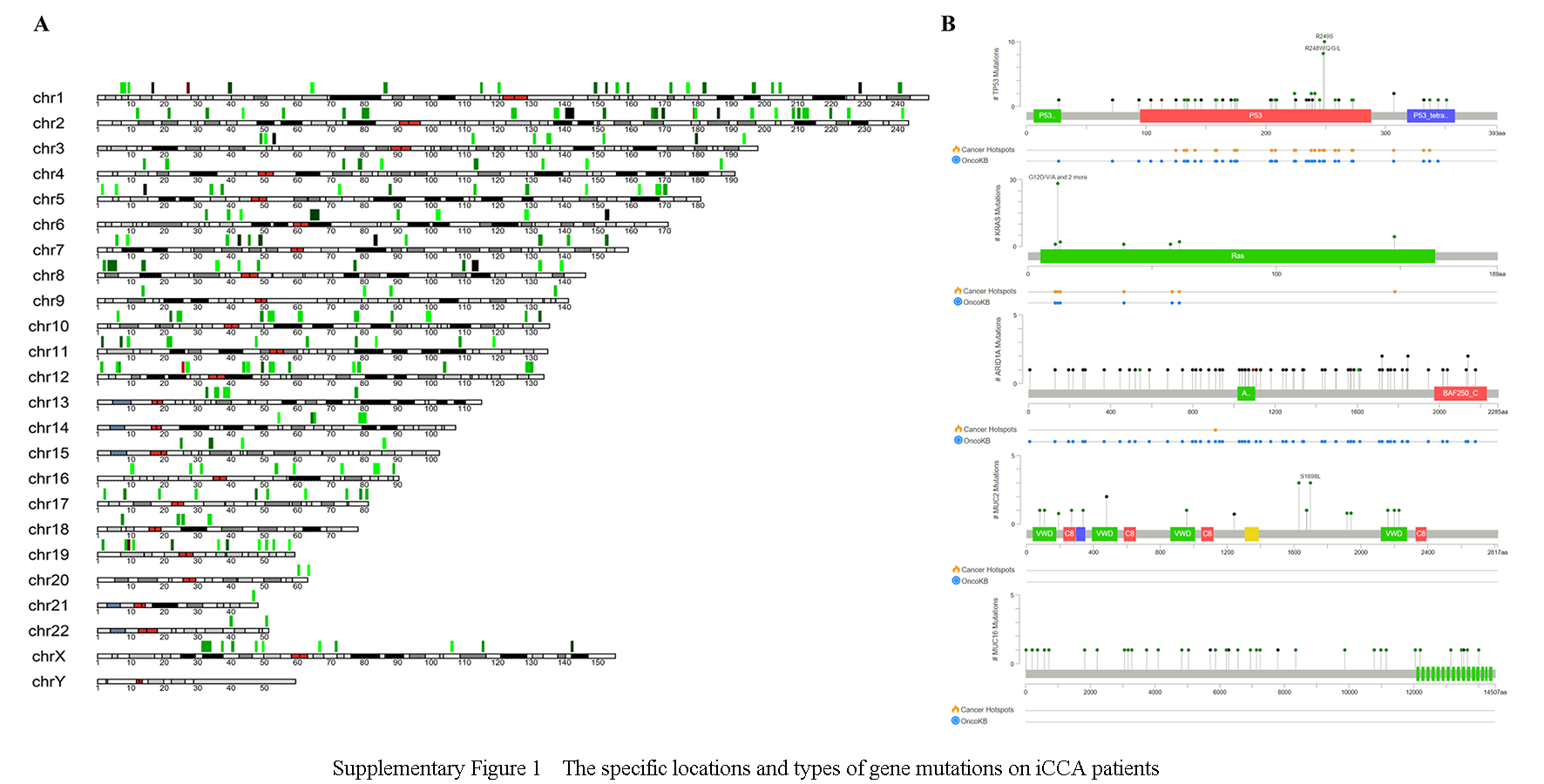

Supplement: Supplementary file 1 — Additional file 1 Figure S1 The specific locations and types of gene mutations on iCCA patients. (a) Map shows specific locations of mutations on chromosomes. Green color indicates low-frequency mutation sites, while red represents high frequency; (b) lollipop plot of top five mutated genes. [file 12885_2021_7788_MOESM1_ESM.tif]

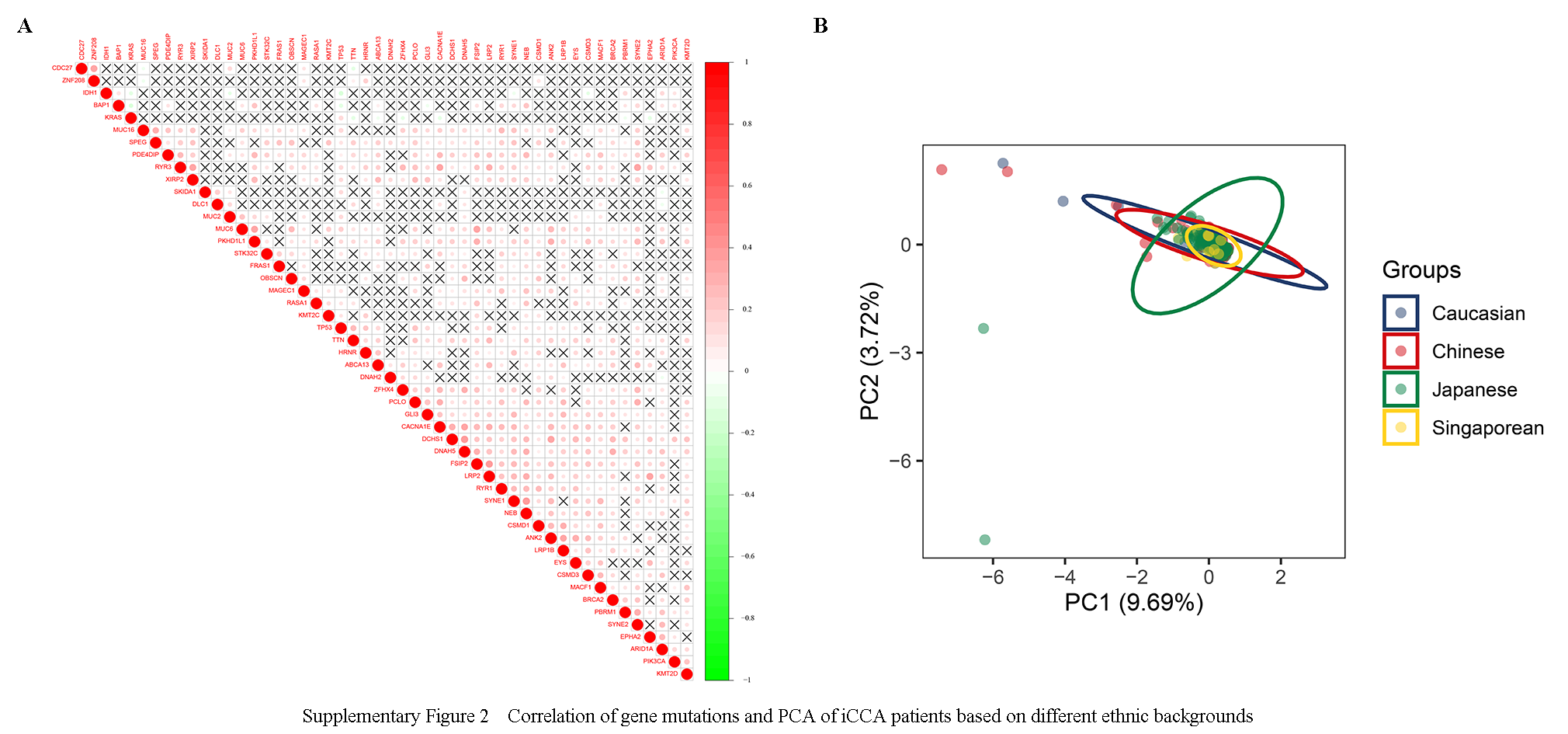

Supplement: Supplementary file 2 — Additional file 2 Figure S2 Correlation of gene mutations and PCA of iCCA patients based on different ethnic backgrounds. (a) Correlation of gene mutations that co-occurred or were exclusive of each other. (b) PCA of iCCA patients based on different ethnic backgrounds. [file 12885_2021_7788_MOESM2_ESM.tif]

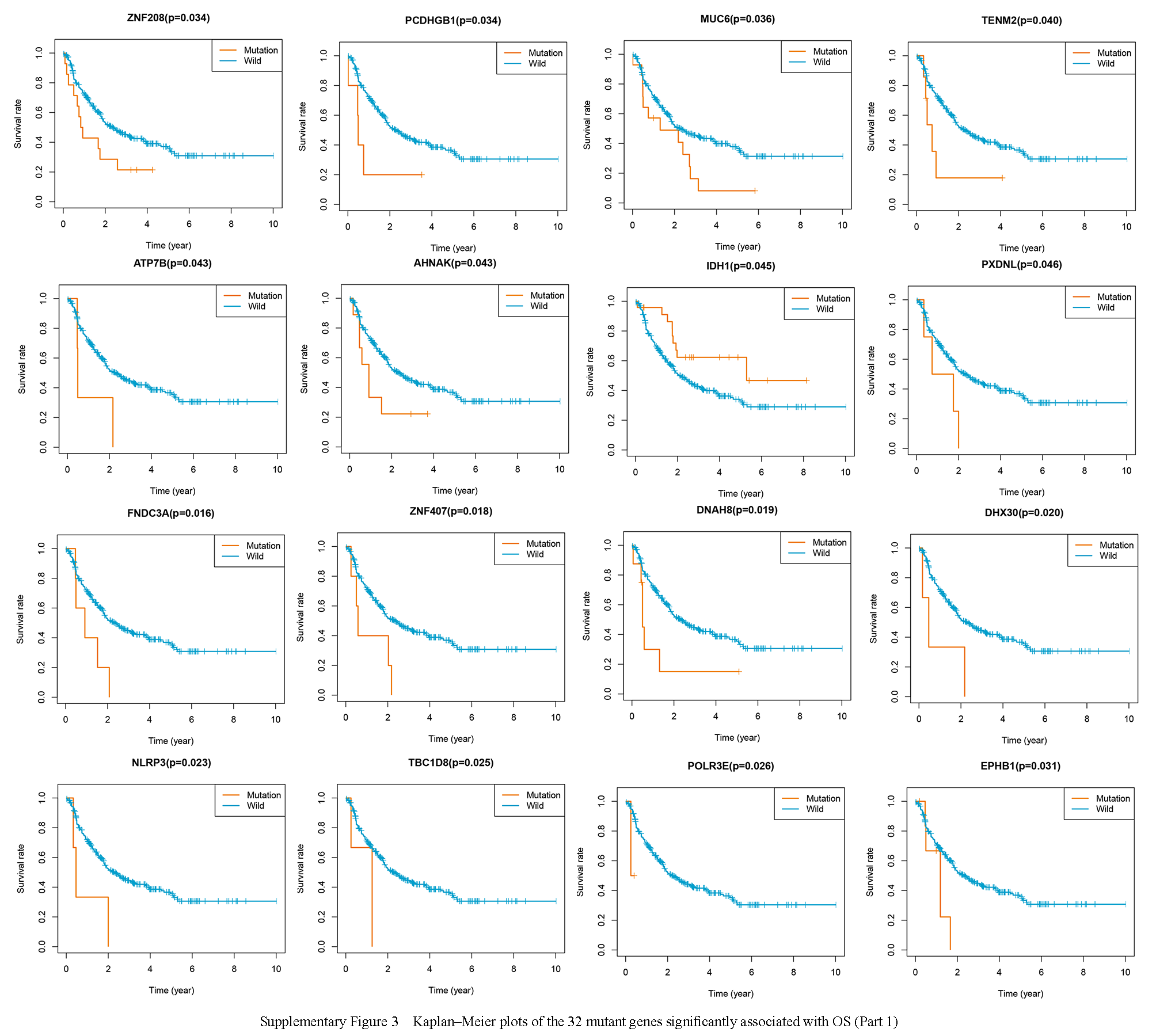

Supplement: Supplementary file 3 — Additional file 3 Figure S3 and Figure S4 Kaplan–Meier plots of the 32 mutant genes significantly associated with OS. [file 12885_2021_7788_MOESM3_ESM.zip › Supplementary Figure 3R3.tif]

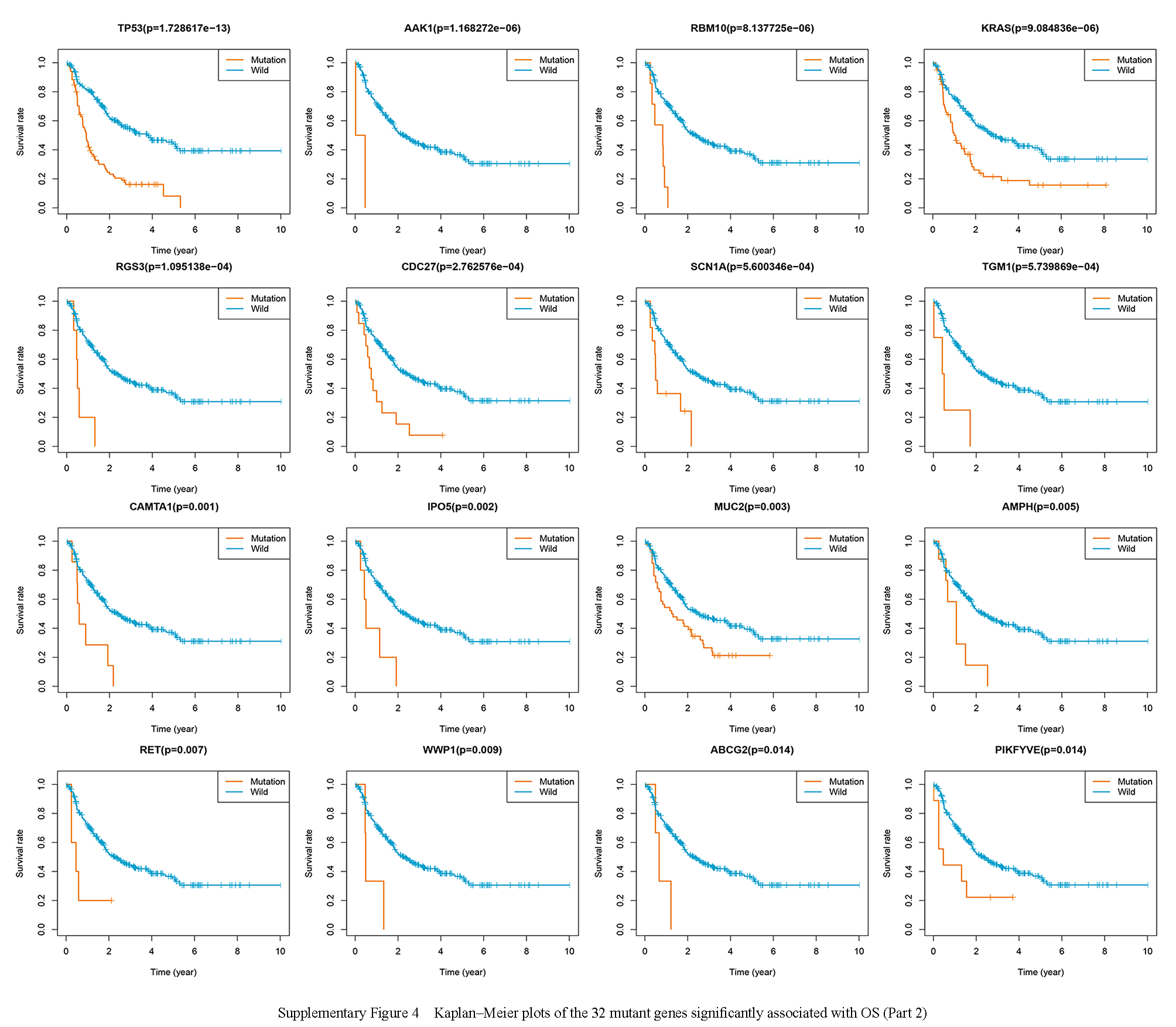

Supplement: Supplementary file 3 — Additional file 3 Figure S3 and Figure S4 Kaplan–Meier plots of the 32 mutant genes significantly associated with OS. [file 12885_2021_7788_MOESM3_ESM.zip › Supplementary Figure 4R3.tif]

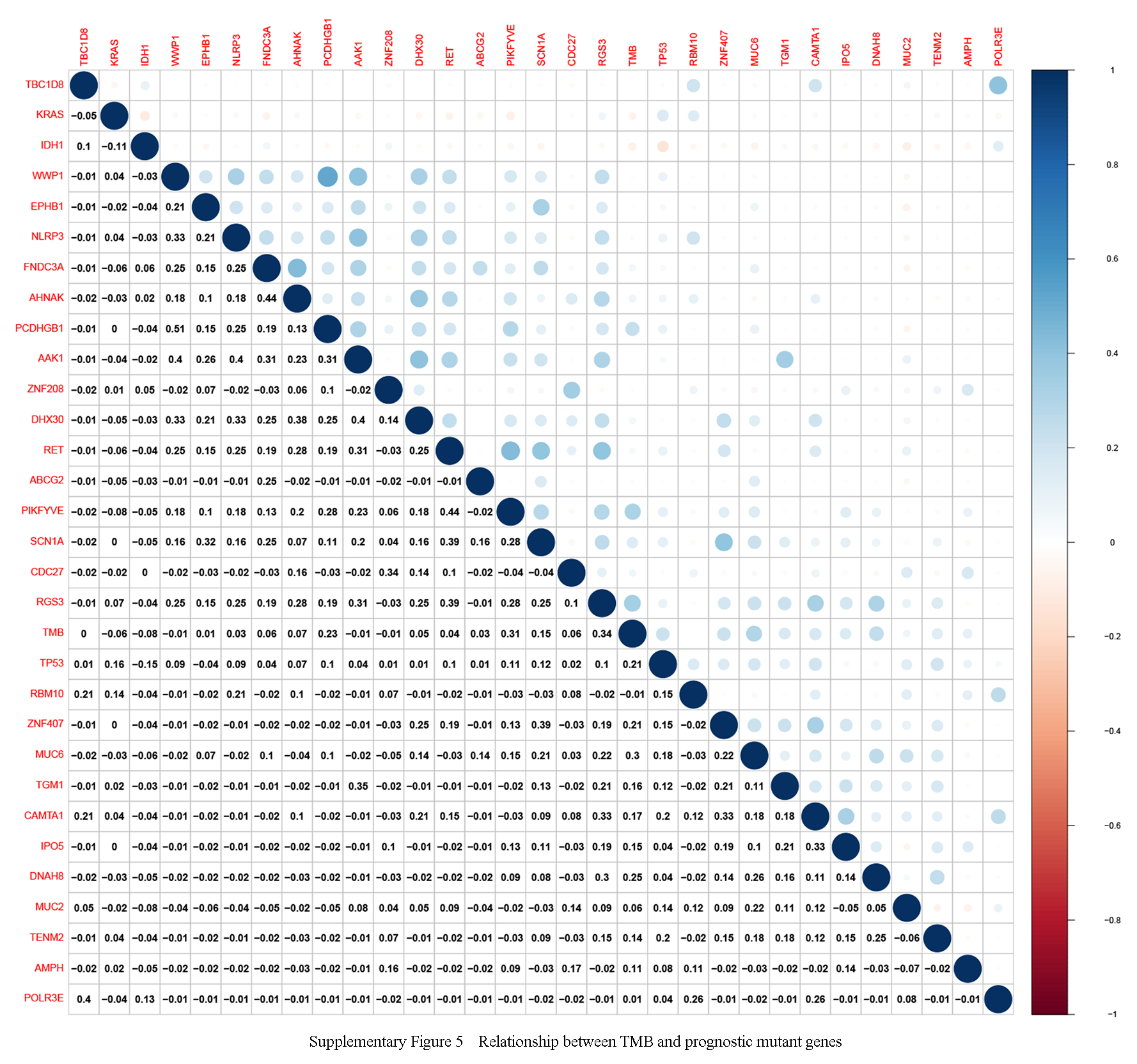

Supplement: Supplementary file 4 — Additional file 4 Figure S5 Relationship between TMB and prognostic mutant genes. [file 12885_2021_7788_MOESM4_ESM.tif]
