# Supplementary material for: Comprehensive analysis of genomic mutation signature and tumor mutation burden for prognosis of intrahepatic cholangiocarcinoma
Source: BMC Cancer. 2021 Feb 3;21:112. doi: 10.1186/s12885-021-07788-7 (PMC7860034; doi:10.1186/s12885-021-07788-7)
Supplement: Supplementary file 5 — Additional file 5. [file 12885_2021_7788_MOESM5_ESM.docx]

Supplementary Table 1: Genetic mutation frequencies in iCCA

| Gene | Frequency | Gene | Frequency | Gene | Frequency | Gene | Frequency |
| --- | --- | --- | --- | --- | --- | --- | --- |
| *TP53* | 85 | *RNF213* | 9 | *UNC5D* | 6 | *MLPH* | 4 |
| *TTN* | 66 | *CACNA1I* | 9 | *CNOT1* | 6 | *KIFC3* | 4 |
| *KRAS* | 61 | *ZNF521* | 9 | *BMP4* | 6 | *SPTAN1* | 4 |
| *MUC2* | 46 | *PIKFYVE* | 9 | *RBM6* | 6 | *SPPL2C* | 4 |
| *ARID1A* | 41 | *FBN3* | 9 | *NRK* | 6 | *PLK3* | 4 |
| *MUC16* | 37 | *BRAF* | 9 | *ROR1* | 6 | *VPS41* | 4 |
| *BAP1* | 29 | *TMEM132D* | 9 | *PTPRK* | 6 | *SCN4A* | 4 |
| *OBSCN* | 27 | *PRKDC* | 9 | *USP29* | 6 | *TTC8* | 4 |
| *CSMD3* | 25 | *ADAMTS20* | 8 | *MAP1B* | 6 | *TNIK* | 4 |
| *EPHA2* | 25 | *SLIT2* | 8 | *UBR2* | 6 | *SEMA4C* | 4 |
| *IDH1* | 24 | *WDR81* | 8 | *AKAP13* | 6 | *LNX1* | 4 |
| *PCLO* | 24 | *CACNA1G* | 8 | *TLE4* | 6 | *PDE3A* | 4 |
| *LRP1B* | 23 | *DNAH8* | 8 | *NXPH1* | 6 | *TGM1* | 4 |
| *PBRM1* | 23 | *NBEA* | 8 | *HEPH* | 6 | *CWH43* | 4 |
| *SYNE1* | 22 | *CNTNAP5* | 8 | *HPSE2* | 6 | *ALPK3* | 4 |
| *DNAH5* | 19 | *MYO15A* | 8 | *ZFP36L2* | 6 | *PDCD11* | 4 |
| *FSIP2* | 18 | *WNK1* | 8 | *PAPPA2* | 6 | *ANO3* | 4 |
| *GLI3* | 18 | *ADAMTS12* | 8 | *KMT2A* | 6 | *SLC9A3* | 4 |
| *EYS* | 17 | *FAM47C* | 8 | *DIDO1* | 6 | *MYO5C* | 4 |
| *PIK3CA* | 17 | *NELL1* | 8 | *KIRREL2* | 6 | *CCDC93* | 4 |
| *LRP2* | 17 | *KIAA1217* | 8 | *SLC10A7* | 6 | *HOOK1* | 4 |
| *MAGEC1* | 17 | *TNRC18* | 8 | *ADAMTS16* | 6 | *IP6K2* | 4 |
| *ABCA13* | 16 | *AMPH* | 8 | *CACNA1F* | 6 | *ROS1* | 4 |
| *MACF1* | 16 | *CELA1* | 8 | *GRIN2A* | 6 | *SPAG4* | 4 |
| *DCHS1* | 15 | *TMEM132C* | 8 | *RBM27* | 6 | *GRM5* | 4 |
| *HRNR* | 15 | *MPDZ* | 8 | *GABRG2* | 6 | *KCNB2* | 4 |
| *KMT2D* | 15 | *ASH1L* | 8 | *PLA2G4C* | 6 | *SPINK5* | 4 |
| *RYR1* | 14 | *PLEKHA6* | 8 | *ARHGAP36* | 5 | *CSE1L* | 4 |
| *MUC6* | 14 | *MDN1* | 8 | *CAMSAP1* | 5 | *SNX14* | 4 |
| *STK32C* | 14 | *PLXNB2* | 8 | *JMJD4* | 5 | *TM9SF3* | 4 |
| *ZNF208* | 14 | *APC* | 8 | *MORC1* | 5 | *FRY* | 4 |
| *PDE4DIP* | 14 | *NAV3* | 8 | *ZNF609* | 5 | *SAGE1* | 4 |
| *RYR3* | 14 | *ERBB4* | 8 | *NLRP5* | 5 | *IRF6* | 4 |
| *CSMD1* | 14 | *KIF19* | 8 | *FNDC3A* | 5 | *BRD2* | 4 |
| *CACNA1E* | 14 | *RABL6* | 8 | *FHOD3* | 5 | *EPHX1* | 4 |
| *CDC27* | 13 | *PLEKHG3* | 8 | *UNC13C* | 5 | *VAV3* | 4 |
| *PKHD1L1* | 13 | *KCNMA1* | 8 | *PLA2G4E* | 5 | *ARGLU1* | 4 |
| *ZFHX4* | 13 | *CHD9* | 8 | *TSC2* | 5 | *ZSCAN12* | 4 |
| *SKIDA1* | 13 | *GREB1* | 8 | *HECW2* | 5 | *MPRIP* | 4 |
| *DLC1* | 13 | *PCDH10* | 7 | *ANKRD62* | 5 | *DSG4* | 4 |
| *XIRP2* | 13 | *DAPK1* | 7 | *OVCH1* | 5 | *HEG1* | 4 |
| *NEB* | 13 | *MYO18A* | 7 | *SDK2* | 5 | *PXDNL* | 4 |
| *SYNE2* | 13 | *ST5* | 7 | *CYP3A5* | 5 | *MGA* | 4 |
| *KMT2C* | 13 | *ARFGAP2* | 7 | *PCDHGB1* | 5 | *DHX9* | 4 |
| *ANK2* | 12 | *ZNF534* | 7 | *SLC9A1* | 5 | *NOS3* | 4 |
| *FRAS1* | 12 | *SYCP2* | 7 | *RET* | 5 | *COL5A1* | 4 |
| *DNAH2* | 12 | *NOTCH2* | 7 | *CHL1* | 5 | *CC2D1B* | 4 |
| *RASA1* | 12 | *KAT6A* | 7 | *ZNF407* | 5 | *CYBB* | 4 |
| *BRCA2* | 12 | *RBM10* | 7 | *SLC12A1* | 5 | *DGKA* | 4 |
| *SPEG* | 12 | *TRPC4* | 7 | *CNTLN* | 5 | *UBE4B* | 3 |
| *NPAP1* | 11 | *PRKG1* | 7 | *CARD11* | 5 | *BCHE* | 3 |
| *BIRC6* | 11 | *KRT85* | 7 | *DSCAML1* | 5 | *MAP2K1* | 3 |
| *CTNNA2* | 11 | *FAT1* | 7 | *RGS3* | 5 | *HEY2* | 3 |
| *ALB* | 11 | *CA6* | 7 | *MEGF10* | 5 | *UBN1* | 3 |
| *VWF* | 11 | *IGSF10* | 7 | *ZNF839* | 5 | *ZFC3H1* | 3 |
| *DNAH17* | 11 | *THSD7B* | 7 | *TRRAP* | 5 | *CCDC85A* | 3 |
| *FMN2* | 11 | *TENM2* | 7 | *AFF1* | 5 | *RIT1* | 3 |
| *PLXNA4* | 11 | *KCNQ3* | 7 | *FOXP1* | 5 | *HCN1* | 3 |
| *ALMS1* | 11 | *COL6A6* | 7 | *ZNF423* | 5 | *DHX30* | 3 |
| *WDFY4* | 11 | *SCN8A* | 7 | *SUPT6H* | 5 | *DYSF* | 3 |
| *MYO7A* | 11 | *ANK3* | 7 | *SLITRK1* | 5 | *GTF2IRD1* | 3 |
| *RBMXL3* | 11 | *CCDC141* | 7 | *HDAC4* | 5 | *RBM12* | 3 |
| *SPTA1* | 11 | *CCDC178* | 7 | *INPPL1* | 5 | *PCSK6* | 3 |
| *UNC80* | 11 | *SLC9C1* | 7 | *ABCC1* | 5 | *STX1A* | 3 |
| *LAMA1* | 11 | *FREM2* | 7 | *KCNH8* | 5 | *TBC1D8* | 3 |
| *COL24A1* | 11 | *CAMTA1* | 7 | *ZNF536* | 5 | *ZNF544* | 3 |
| *NACAD* | 11 | *GTF3C1* | 7 | *CDH7* | 5 | *ABCG2* | 3 |
| *SCN1A* | 11 | *SRCAP* | 7 | *SPTBN1* | 5 | *NLRP3* | 3 |
| *COL7A1* | 11 | *DOCK10* | 7 | *BCAP29* | 5 | *KLHL11* | 3 |
| *APOB* | 11 | *EPHB1* | 7 | *GIGYF2* | 5 | *ZDHHC17* | 3 |
| *KCNT2* | 11 | *NTF3* | 7 | *ZNF14* | 5 | *FMN1* | 3 |
| *PRRC2C* | 10 | *LRRD1* | 7 | *CSPG4* | 5 | *ANGPT1* | 3 |
| *ATM* | 10 | *PCNT* | 7 | *RAD21* | 5 | *PIK3CB* | 3 |
| *STK11* | 10 | *TYK2* | 7 | *CDHR2* | 5 | *CCBE1* | 3 |
| *BCOR* | 10 | *MYH14* | 7 | *HMGCR* | 5 | *SENP2* | 3 |
| *PTEN* | 10 | *DNAH7* | 7 | *BRWD1* | 5 | *KATNAL2* | 3 |
| *LRP1* | 10 | *FAM208B* | 7 | *WNK4* | 5 | *PPP2R3A* | 3 |
| *STAB2* | 10 | *NRXN3* | 7 | *DIAPH3* | 5 | *ATP7B* | 3 |
| *NIPBL* | 10 | *SNED1* | 6 | *USP34* | 5 | *NOD2* | 3 |
| *DOCK2* | 10 | *WDFY3* | 6 | *NID1* | 5 | *TET3* | 3 |
| *NRAS* | 10 | *NBEAL1* | 6 | *TRIP11* | 5 | *WWP1* | 3 |
| *MED12* | 10 | *MED13* | 6 | *MAST4* | 5 | *EXTL3* | 3 |
| *DLGAP2* | 10 | *GRIK2* | 6 | *TSC1* | 5 | *AAK1* | 2 |
| *NOTCH4* | 10 | *ITPR2* | 6 | *MRC2* | 5 | *PJA1* | 2 |
| *FBN2* | 10 | *NELL2* | 6 | *IPO5* | 5 | *POLR3E* | 2 |
| *DMD* | 10 | *ZFHX3* | 6 | *MTUS2* | 5 | *TRIML2* | 2 |
| *MYCBP2* | 10 | *OSBPL8* | 6 | *SCN7A* | 5 | *OGDHL* | 2 |
| *ZNF469* | 9 | *COL22A1* | 6 | *EML6* | 5 | *ZFR* | 2 |
| *CCDC88A* | 9 | *CDH13* | 6 | *MYH7B* | 5 | *DOCK7* | 2 |
| *BOD1L1* | 9 | *UBR1* | 6 | *ITPR3* | 5 | *CRHR1* | 2 |
| *ELF3* | 9 | *PCF11* | 6 | *DCLK1* | 4 | *GFM1* | 2 |
| *LAMA3* | 9 | *ATP13A4* | 6 | *ARHGEF38* | 4 | *RNF103* | 2 |
| *MKI67* | 9 | *NKD2* | 6 | *C6orf10* | 4 | *NAV1* | 1 |
| *AHNAK* | 9 |  |  | *PRG4* | 4 |  |  |
